# Adult Learning and Language Simplification

**DOI:** 10.1111/cogs.12686

**Published:** 2018-10-15

**Authors:** Mark Atkinson, Kenny Smith, Simon Kirby

**Affiliations:** ^1^ Department of Psychology University of Stirling; ^2^ School of Philosophy, Psychology and Language Sciences University of Edinburgh

**Keywords:** Language evolution, Language complexity, Cultural transmission, Adult learning, Linguistic accommodation, Foreigner‐directed speech

## Abstract

Languages spoken in larger populations are relatively simple. A possible explanation for this is that languages with a greater number of speakers tend to also be those with higher proportions of non‐native speakers, who may simplify language during learning. We assess this explanation for the negative correlation between population size and linguistic complexity in three experiments, using artificial language learning techniques to investigate both the simplifications made by individual adult learners and the potential for such simplifications to influence group‐level language characteristics. In Experiment 1, we show that individual adult learners trained on a morphologically complex miniature language simplify its morphology. In Experiment 2, we explore how these simplifications may then propagate through subsequent learning. We use the languages produced by the participants of Experiment 1 as the input for a second set of learners, manipulating (a) the proportion of their input which is simplified and (b) the number of speakers they receive their input from. We find, contrary to expectations, that mixing the input from multiple speakers nullifies the simplifications introduced by individuals in Experiment 1; simplifications at the individual level do not result in simplification of the population's language. In Experiment 3, we focus on language *use* as a mechanism for simplification, exploring the consequences of the interaction between individuals differing in their linguistic competence (as native and non‐native speakers might). We find that speakers who acquire a more complex language than their partner simplify their language during interaction. We ultimately conclude that adult learning can result in languages spoken by more people having simpler morphology, but that idiosyncratic simplifications by non‐natives do not offer a complete explanation in themselves; accommodation—by comparatively competent non‐natives to less competent speakers, or by native speakers to non‐natives—may be a key linking mechanism.

## 1 Introduction

Languages are products of cultural evolutionary processes, transmitted across generations of users via social learning. Because they are transmitted in this way, they have been repeatedly subjected to the pressures of learning and use, and this is reflected in their structure (Beckner et al., 2009; Christiansen & Chater, 2008; Croft, 2000; Smith & Kirby, 2008). It has been suggested that these pressures might differ in different physical, demographic, and sociocultural environments, and that such non‐linguistic factors could therefore systematically shape linguistic features (Croft, 1995; Dale & Lupyan, 2012; Nettle, 1999; Trudgill, 2011; Wray & Grace, 2007). Identifying the factors which probabilistically influence language structure, along with the mechanisms by which they operate, would provide insights into the causes and limits of linguistic diversity (Dale & Lupyan, 2012; Wray & Grace, 2007) and would allow us to build a picture of the characteristics of the earliest languages of our species based on assumptions about the environments in which they were spoken (Wray & Grace, 2007).

A growing body of work seeks to explain properties of linguistic systems in terms of their social environments. We review this literature below, focussing on a prominent group of related theories which link linguistic complexity to social structure, in particular population size and the proportion of adult learners in a population. Several authors claim, supported by circumstantial evidence, that languages spoken in large populations featuring a relatively high proportion of non‐native speakers are simpler than languages spoken in smaller populations where non‐native speakers are rare. While we find these claims intriguing and plausible, direct experimental tests of the proposed links between linguistic complexity, population size, and proportion of non‐native speakers are lacking. We therefore test these proposed mechanisms by which size or composition of a population might influence the complexity of that population's language. In Experiment 1, we use an artificial language learning paradigm to test whether learners tend to simplify languages during the early stages of learning, and we find that they do. In Experiment 2, we use similar methods to test whether the propagation of the simplifications through subsequent learning is dependent on population size and the proportion of non‐native‐like simplifying speakers. Perhaps surprisingly, we find that simplifications do not propagate under any of the conditions we investigated, casting doubt on accounts which assume a straightforward link between adult learning and language simplification. Finally, in Experiment 3, we show how interaction could in principle provide such a linking mechanism: simplifications of the kind made by non‐native learners are preferentially adopted during interaction and are therefore more likely to spread.

### 1.1 The influence of social factors on language structure

Previous work has investigated how elements of the physical environment, biology, society, and culture may influence cross‐linguistic variation, and it studied their effect on a range of different language features (see Everett, Blasi, & Roberts, 2015; Regier, Carstensen, & Kemp, 2016, for recent examples). Here we focus on a growing body of work that looks at the influence of sociocultural factors on cross‐linguistic variation in language complexity (Nettle, 2012; Trudgill, 2011). Defining complexity is contentious, but traits such as irregularities, opaque forms, holism, suppletion, and presence of certain morphological features (e.g., morphological encoding of evidentiality or past tense remoteness distinctions) are typically taken as indicative of complexity. Trudgill (2011), Thurston (1994), and Wray and Grace (2007) each suggest that languages primarily employed for intra‐group communication (*esoteric* languages) will be more complex than those typically used for inter‐group communication (*exoteric* languages). Esoteric language use is associated with limited language contact, social stability, small group size, dense social networks, and large amounts of communally shared information, and esoteric languages are claimed to be characterized by complex language features and lower levels of expressive flexibility (i.e., reduced potential to produce novel utterances which can be interpreted without substantial scaffolding from the non‐linguistic context). Trudgill (2011), for example, highlights the development of Afrikaans as a simplified variant of Dutch as a result of contact and demographic factors, acquiring lasting simplifications as a result of children acquiring the language from non‐native adult speakers. In the comparatively isolated case of Faroese, by contrast, the complexity of its deictic system and degree of morphological opacity has increased over time, uninfluenced by non‐native learners.

Analyses of large datasets offer at least partial support for these claims. Following smaller studies which found statistical relationships between morphological complexity and number of speakers (Nichols, 2009; Sinnemäki, 2009), Lupyan and Dale (2010) investigate 28 structural features taken from the World Atlas of Language Structures (Dryer & Haspelmath, 2013), relating to morphological type, case system, verbal morphology, agreement, possibility and evidentials, negation, plurality, interrogatives, tense, possession, aspect, mood, articles, demonstratives, and pronouns. From a sample of 2,236 languages, they conclude that morphological complexity is inversely correlated to number of speakers, the area a language is spoken over, and the number and type of neighboring languages. Though this relationship holds whichever variable is considered, population size has the greatest predictive power. As reviewed below, adult learning has been widely proposed as a factor in the simplification of language (Dahl, 2004; Kusters, 2003; McWhorter, 2005, 2007; Nettle, 2012; Trudgill, 2004, 2011; Wray & Grace, 2007); Lupyan and Dale (2010) (also Dale & Lupyan, 2012) suggest their results can therefore be explained as a consequence of adult learning, on the assumption that languages with greater numbers of speakers are also those with greater proportions of adult learners as a result of contact with speakers of other languages. This would suggest that the proportion of non‐native speakers should be a more direct predictor of linguistic complexity than absolute number of speakers (Nettle, 2012). If so, we may expect that languages spoken by smaller numbers of speakers, but which have higher proportions of non‐native speakers, will have lower levels of linguistic complexity; conversely, languages spoken by larger groups, but with relatively few non‐native speakers, will be more complex. Bentz and Winter (2013) offer some support for this by demonstrating that case marking is negatively correlated with the proportion of second language learners; languages in which the majority of the speakers are second language speakers are those which have no case marking.

### 1.2 Adult learning and simplification

Why would the languages of larger populations, where exoteric communication is the norm and adult learners are common, be simpler? In other works, we have explored the impact of input variability and esotericity on language learning and language use (Atkinson, Kirby, & Smith, [Link]; Atkinson, Mills, & Smith, in press^2015^), and find little evidence of an effect of either raw input variability (likely to characterize learning in larger or more exoteric populations) or shared knowledge (likely to characterize esoteric communication) on linguistic complexity. In this paper we look at the role of adult learners in reducing linguistic complexity. At the individual level, there is a clear relationship between age of acquisition and ultimate language proficiency, and strong evidence to suggest that learning a language after puberty leads to productive and receptive deficiencies in phonology, morphology, and syntax (Clahsen, Felser, Neubauer, Sato, & Silva, 2010; Clark, 2003; Johnson & Newport, 1989; Kusters, 2003; Lenneberg, 1967; McWhorter, 2007; Newport, 1990, 2002; Scovel, 2000; Trudgill, 2011). Ultimate attainment is also highly variable for older learners, dependent on age of acquisition, learning context, and learner motivation (Bley‐Vroman, 1989; Csizér & Dörnyei, 2005; Nettle, 2012; Selinker, 1972). Adult learners are thought to find certain linguistic features particularly challenging to acquire, including morphological complexity, syntagmatic and paradigmatic redundancy, and irregularities, even when similar features are found in their native language (Bentz & Winter, 2013; Clahsen et al., 2010; Lupyan & Dale, 2010; Trudgill, 2011; Wray & Grace, 2007). A number of studies provide empirical support. For example, Klein and Perdue (1997) describe a longitudinal study of adult learners of Dutch, English, French, German, and Swedish, whose productions were found to be limited in terms of case, number, gender, tense, aspect, and morphological agreement: “lexical items typically occur in one invariant form … [though if] a word shows up in more than one form, … this (rare) variation does not seem to have any functional value: the learners simply try different phonological variants” (Klein & Perdue, 1997, p. 311); Parodi, Schwartz, and Clahsen (2004) illustrate that adult learners of German find morphological inflections challenging regardless of their native language, while English (Gürel, 2000; Haznedar, 2006) and Greek (Papadopoulou et al., 2011) learners of Turkish find case particularly difficult, with suffixes generally omitted in production. Zero‐marking is not the only means by which adult learners produce simplified forms, there is also overgeneralization of “a form which would be an inflected form in the target language” (Klein & Perdue, 1997, p. 311).

There are notable exceptions to this general consensus that adult learners tend to produce simplified linguistic systems, however; Cuskley et al. (2015) found that non‐native speakers were more likely than native speakers to produce irregular forms when asked to produce the past‐tense form of a novel verb. As the authors note, this may not necessarily imply that non‐natives will increase the complexity of the overall past‐tense system, but this study at the very least demonstrates that non‐native realization of linguistic rules warrants further investigation.

Under these accounts, languages with greater degrees of adult learning are under increased pressure for simplification due to these acquisition difficulties, even if “[t]he evidence for such linguistic simplification has largely been descriptive, consisting of selected examples and grammatical inventories of small numbers of languages” (Lupyan & Dale, 2010, p. 2). According to this hypothesis, the languages adapt to the needs and abilities of the older learners, with the more “difficult” features filtered out (Bentz & Winter, 2013; Lupyan & Dale, 2010; Wray & Grace, 2007). Many of these features may be informationally redundant anyway, in that the information which is no longer obligatorily encoded in the linguistic signal (e.g., situational/epistemic possibility, evidentiality, indefiniteness, distance contrasts in demonstratives, remoteness distinctions in the past tense) is retrievable from either linguistic or pragmatic context, and so such languages can comfortably tolerate these lower levels of complexity (Dahl, 2004; Gil, 2009; Lupyan & Dale, 2010). This reduction in complexity will result in a greater reliance on extralinguistic, pragmatic, information, but again it is claimed that this better suits adult learners than children (Lupyan & Dale, 2010). Conversely, Lupyan and Dale (2010) also speculate that redundancy may actually aid child learning, in that it provides infants with additional linguistic cues to supplement their relatively undeveloped abilities to use extralinguistic, pragmatic, information (Snedeker & Trueswell, 2004; Trueswell, Sekerina, Hill, & Logrip, 1999; Weighall, 2008). If so, more complex languages might reflect adaptations to the needs of child learners, in the same way that simpler languages reflect adaptations to adult learning.

### 1.3 The problem of linkage

Even if adult learners do simplify morphological systems, this is not a complete explanation for how the presence of adult learners leads to language simplification. There is a problem of linkage: We need to identify some mechanism by which this individual‐level simplification affects language at the level of the population (Kirby, 1999). Even if the children of non‐native learners acquire the simpler language of their parents rather than that of the wider speech community; for example, the simplifications will remain restricted to a subset of the population, rather than having an influence on the language as a whole. We therefore need to explain how such simplifications could spread to reduce complexity at the language level.

When exposed to artificial languages exhibiting unpredictably variable elements (e.g., unconditioned variation in the form of a post‐nominal particle or in word order), children (and to a lesser extent adults) have a tendency to reduce or eliminate that variability (Culbertson & Newport, 2015; Culbertson, Smolensky, & Legendre, 2012; Hudson Kam, 2015; Hudson Kam & Newport, 2005, 2009; Smith & Wonnacott, 2010; Wonnacott, 2011); there is some evidence that younger children similarly fail to capture the full range of sociolinguistically conditioned variation during natural language learning in some circumstances (Habib, 2016; Smith, Durham, & Richards, 2013). If this tendency to eliminate variability applies when learners receive a mix of native and non‐native speaker input, then the cross‐generational transmission of the complex or simple variants may depend on their frequency in the input, or sociolinguistic factors (such as the relative importance for language acquisition of each of the different individuals who provide the input, Barton & Tomasello, 1994), and transmission may result in simplifications propagating once those simplifications achieve critical mass in the input for the next generation of learners.

In Experiments 1–2, we therefore use an artificial language learning paradigm to investigate whether simplifications produced by imperfect learning tend to spread when languages are transmitted. In Experiment 1, we test the ability of adult participants to learn and reproduce a morphologically complex artificial language. Our results here support the claim that adults reduce morphological complexity during the early stages of learning. We then use the languages produced by these participants as input data for a second set of learners in Experiment 2, to test whether these simplifications preferentially spread during subsequent learning. In Experiment 3, we explore whether communicative interaction, rather than learning and reproduction, might constitute an additional or alternative mechanism explaining how simplifications made by adult learners spread at the expense of more complex alternatives.

## 2 Experiment 1: Adult learning and morphological simplification

In our first experiment, we trained adult learners on an artificial language providing descriptions for simple scenes. The target language our participants attempted to learn exhibited morphological complexity, in that suffixes redundantly mark number (singular vs. plural) and do so irregularly. This first experiment allows us to assess the claim that non‐native speakers will acquire and produce a simpler morphological system than that of their target language. It also provides a set of linguistic stimuli exhibiting natural variation in acquisition fidelity, which will be used in Experiment 2.

### 2.1 Method

#### 2.1.1 Participants

In this study, 26 native English speakers (15 female, 11 male; aged between 18 and 29, *M = *21.6) were recruited using the Student and Graduate Employment (SAGE) database of the Careers Service at the University of Edinburgh. Each participant was compensated £7.

#### 2.1.2 The target language

Participants were trained on an artificial language which provides descriptions for scenes involving animals performing movements. Each scene features either one or two animals of the same type (one or two crocodiles, one or two ducks, or one or two birds), marked with arrows to indicate three different motions (straight motion, bouncing, or looping). The full set of images is given in Supplemental Information S1.

Each image is uniquely described by three words, with (the artificial language equivalents of) quantifiers to describe Number, nouns to describe Animal, and verbs to describe Motion. Each individual word is made up of a stem and a suffix. The quantifier stems are *won* (“one” for 1) and *sum* (“some” for 2); the noun stems are *snap* (crocodile), *kwak* (duck), and *twit* (bird); the verb stems are *woosh* (straight motion), *boing* (bouncing), and *loop* (looping). The stems were designed to be transparent in meaning and easily learned by English‐speaking participants (following Culbertson & Newport, 2015; Fehér, Wonnacott, & Smith, 2016); we avoided using actual English words in all the labels (as in Smith & Wonnacott, 2010) in order to make the existence of non‐English suffixes in the artificial language less unexpected. Our intention was that, by using these easily acquired stems, participants would be able to produce sets of labels which unambiguously identified all of the images after only limited training, even if the stems were not acquired with complete accuracy, and regardless of how well the suffixes had been learned. This allows us to focus on the learning of the morphological systems expressed by the suffixes. The suffixes mark number on all three lexical items and include a mix of regular and irregular forms—see the full language in Table 1. In the table, the suffixes are separated from the stem by a hyphen for clarity, although these hyphens were not included in the actual strings shown to participants. For the quantifiers, *‐a* and *‐ak* mark singular and plural images of ducks and birds; exceptionally, the quantifiers for crocodile mark singular and plural with *‐u* and *‐uk*. For the nouns, *‐o* and *‐op* mark singular and plural, except for birds, where *‐o* is used for both singular and plural. For the verbs, *‐an* and *‐asp* are used for singular and plural for the duck and bird, while *‐en* and *‐esp* are used for the crocodile; an additional exception arises where the looping motion occurs with a plural image, in which case all verbs take the *‐onk* suffix. As discussed in the Introduction, such informationally redundant language features are specifically thought to be challenging for non‐native speakers to acquire (Lupyan & Dale, 2010; Trudgill, 2011; Wray & Grace, 2007), with experimental evidence that morphological complexity like this will typically result in a speaker omitting the affix altogether or using a single invariant form (Haznedar, 2006; Klein & Perdue, 1997; Parodi et al., 2004).

**Table 1:** Target language

Description in the Target Language			English Gloss
won‐a	kwak‐o	woosh‐an	“one duck straight”
sum‐ak	kwak‐op	woosh‐asp	“two ducks straight”
won‐a	kwak‐o	boing‐an	“one duck bounce”
sum‐ak	kwak‐op	boing‐asp	“two ducks bounce”
won‐a	kwak‐o	loop‐an	“one duck loop”
sum‐ak	kwak‐op	loop‐onk	“two ducks loop”
won‐a	twit‐o	woosh‐an	“one bird straight”
sum‐ak	twit‐o	woosh‐asp	“two birds straight”
won‐a	twit‐o	boing‐an	“one bird bounce”
sum‐ak	twit‐o	boing‐asp	“two birds bounce”
won‐a	twit‐o	loop‐an	“one bird loop”
sum‐ak	twit‐o	loop‐onk	“two birds loop”
won‐u	snap‐o	woosh‐en	“one crocodile straight”
sum‐uk	snap‐op	woosh‐esp	“two crocodiles straight”
won‐u	snap‐o	boing‐en	“one crocodile bounce”
sum‐uk	snap‐op	boing‐esp	“two crocodiles bounce”
won‐u	snap‐o	loop‐en	“one crocodile loop”
sum‐uk	snap‐op	loop‐onk	“two crocodiles loop”

#### 2.1.3 Procedure

Participants were trained and tested on the target language over eight rounds, each round consisting of a training phase and a testing phase.

In a *training phase,* 9 of the 18 stimuli and their descriptions were randomly selected as training items (this random selection step was repeated for each training phase, and so the training items were unlikely to be the same from one training phase to the next). The participant was then trained on five randomly sorted passes of this set. On each training trial, the image was presented for 1 s, and then the image and description (presented as text) were presented together for a further 5 s. The description was then removed, and the participant was required to retype the description from memory. No feedback was given as to the accuracy of the retyping.

In the *testing phase* following each training phase, the participant was presented with all 18 images in a random order and required to provide a description for each; on each trial, they were simply prompted with an image and required to type in the appropriate description in the artificial language, with no feedback provided regarding accuracy. Note that participants were able to provide any description during testing; there was nothing to restrict their use of, for example, suffixes they had not encountered for a given word type in training, or dropping suffixes altogether.

The experiment was written and run in Matlab (R2010a) with the Psychtoolbox extensions (Brainard, 1997; Kleiner, Brainard, & Pelli, 2007).

### 2.2 Results

#### 2.2.1 Acquisition of stems and suffixes

**Figure 1:**
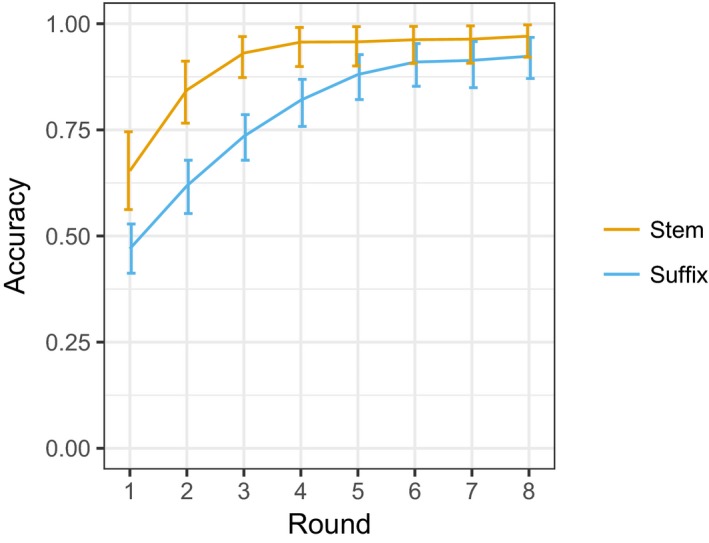
Average stem and suffix accuracy by round. Accuracy scores for a given image calculated as 1 minus the normalized Levenshtein distance between the target description and the description produced by the participant, and we plot the mean of the by‐participant mean accuracy; error bars indicate 95% confidence intervals on this mean of means. Stems are more accurately reproduced than suffixes, and accuracy increases over rounds for both stems and suffixes.

For each round, the stems and suffixes in the productions were separated: Where the start of a word exactly matched a stem from the target language, this was done automatically; otherwise this was done by hand. Zero‐marked suffixes were coded as NULL for analysis purposes. The difference between the target stem or suffix and the stem or suffix produced by the participant was measured using normalized Levenshtein distance,^1^ and accuracy was defined as 1 minus this distance. These accuracy scores are illustrated in Fig. 1.

Stem and suffix accuracy were both significantly greater in Round 8 compared to Round 1( | *t*(25) | ≥ 6.861, *p* < .001). Stem accuracy is close to ceiling from Round 4 onwards (average ≥ 0.96). This is unsurprising given the design of the language. Suffix accuracy is lower, though the productions are close to the target language suffixes from Round 6 onwards (average ≥ 0.91).

#### 2.2.2 Complexity of conditioning

To quantify the complexity of such suffix sets, we consider the complexity of the relationship between semantic features of the stems and the suffixes used. In Supplemental Information S2, we report an additional meaning‐independent measure, the entropy of the suffixes.

To assess whether adult learners simplify morphology, we compare their suffix productions across rounds. Our complexity measure can be applied to the data produced by participants in the test phase in each of the eight rounds of the experiment, but of particular relevance for Experiment 2 will be the contrast between Round 2, where participants have generally acquired the stems of the language but have yet to acquire the suffixes accurately, and those of Round 8, where they have acquired or are close to acquiring the target language.^2^ Two example sets of suffixes, produced by the same participant in Round 2 and Round 8, are shown in Table 2. The stem productions for both these languages exactly matched those of the target language (stem accuracy = 1). By Round 8, the participant has perfectly acquired the target language (suffix accuracy = 1). The Round 2 language would appear to be simpler than the Round 8 language. The quantifier suffixes, for example, are entirely conditioned on Number in Round 2. In the Round 8 example, on the other hand, they are also dependent on Animal, as in the target language.

**Table 2:** Example Round 2 and Round 8 suffix sets produced by Participant 2

	Round 2			Round 8		
	Q	N	V	Q	N	V
“one duck straight”	‐a	‐o	‐esp	‐a	‐o	‐an
“two ducks straight”	‐ak	‐op	‐esp	‐ak	‐op	‐asp
“one duck bounce”	‐a	‐o	‐esp	‐a	‐o	‐an
“two ducks bounce”	‐ak	‐op	‐esp	‐ak	‐op	‐asp
“one duck loop”	‐a	‐o	‐an	‐a	‐o	‐an
“two ducks loop”	‐ak	‐op	‐an	‐ak	‐op	‐onk
“one bird straight”	‐a	‐o	‐esp	‐a	‐o	‐an
“two birds straight”	‐ak	‐op	‐esp	‐ak	‐o	‐asp
“one bird bounce”	‐a	‐o	‐esp	‐a	‐o	‐an
“two birds bounce”	‐ak	‐o	‐asp	‐ak	‐o	‐asp
“one bird loop”	‐a	‐o	‐an	‐a	‐o	‐an
“two birds loop”	‐ak	‐op	‐an	‐ak	‐o	‐onk
“one crocodile straight”	‐a	‐o	‐esp	‐u	‐o	‐en
“two crocodiles straight”	‐ak	‐op	‐esp	‐uk	‐op	‐esp
“one crocodile bounce”	‐a	‐o	‐esp	‐u	‐o	‐en
“two crocodiles bounce”	‐ak	‐op	‐esp	‐uk	‐op	‐esp
“one crocodile loop”	‐a	‐o	‐an	‐u	‐o	‐en
“two crocodiles loop”	‐ak	‐op	‐an	‐uk	‐op	‐onk

Note

The Round 2 suffix set appears to be simpler than that of Round 8. The quantifier (Q) suffixes are entirely conditioned on Number, with *‐a* for singular and *‐ak* for plural. The noun (N) suffixes are also conditioned on Number, with *‐o* for singular and *‐op* for plural, bar the exception for the scene involving two bouncing birds. The verb (V) suffixes are conditioned on Movement, with straight and bouncing motion taking *‐esp* and looping motion taking *‐an*, bar the exception for two bouncing birds. The Round 8 set is relatively complex. Both the Q and N suffixes are conditioned on both Number and Animal, while the V suffixes are conditioned on Number, Animal, and Movement. Note that in these examples, the participants produced a suffix which they had been exposed to in training for each word type although this was not always the case; other participants produced entirely novel suffixes, or zero‐marking.

The key difference between the simple and complex languages laid out in Table 2 is in the complexity of the conditioning contexts governing which suffix is used when, for the simple language, or the simpler suffixes, this conditioning is based on fewer semantic features (e.g., it depends only on number), whereas for the more complex cases it depends on a combination of factors (e.g., in the target language, the choice of verbal suffix depends on all three features of the scene being described, i.e., the animal, the number, and the motion). There are several ways in which this complexity of conditioning could be captured; here, we use a procedure which involves identifying the best‐fitting statistical model for each suffix type for each participant and evaluating how many predictors that model uses to predict suffix choice—the number of predictors is our measure of complexity.

In more detail: for a given participant's data for a given suffix type (Q, N or V) at a given round, we use a multinomial regression (via the multinom function in R, Venables & Ripley, 2002) to predict suffix choice based on various possible semantic features. We run all possible models featuring all combinations of semantic features (the number, animal or movement in the scene being described) and their interactions,^3^ and then select the model with the lowest Akaike Information Criterion value as the best‐fitting model. In other words, for a given participant's data on a given stem type (Q, N or V) we attempt to predict their suffix choice given all possible combinations of number, animal, and motion, and then select the model which best predicts their data. In order to allow for the fact that some languages collapse across levels of semantic features (e.g., the target language treats duck and bird as equivalent for the purposes of determining quantifier suffix), we include in our set of candidate predictors both the full and collapsed versions of each semantic feature (e.g., for the animal feature, we consider models featuring a three‐way contrast between duck, bird and crocodile, as well as models featuring a two‐way contrast between crocodiles and ducks/birds, between birds and ducks/crocodiles, and between ducks and birds/crocodiles). We then evaluate the complexity of the best‐fitting model, by counting how many semantic features it includes: this complexity score ranges from 0 for models which include only an intercept, for example, if the participant only uses a single suffix, to 3 for participants whose data is best explained by a model which refers to number, animal and motion. The best‐fitting models for the example data in Table 2 are shown in Table 3.^4^

**Table 3:** Model‐based approach to complexity for the example participant data from Table 2

Round	Suffix Type	Best Fitting Model	Model Complexity
2	Q	Suffix ~ Number	1
2	N	Suffix ~ Number + Animal_bird_ + Movement_bounce_	3
2	V	Suffix ~ Movement_loop_	1
8	Q	Suffix ~ Number + Animal_crocodile_	2
8	N	Suffix ~ Number + Animal_bird_	2
8	V	Suffix ~ Number + Animal_crocodile_ + Movement_loop_	3

Notes

The best‐fitting models for the example participant data. For each suffix we show the model which had the lowest AIC in a multinomial regression. Subscripts indicate that the relevant semantic dimension was split into two, differentiating the subscripted value from all others: for example, Suffix ~ Movement_loop_ indicates that the best‐fitting model for predicting suffix choice featured a predictor based on the movement pictured in the scene, where that predictor differentiated looping motion from the other motions.

**Figure 2:**
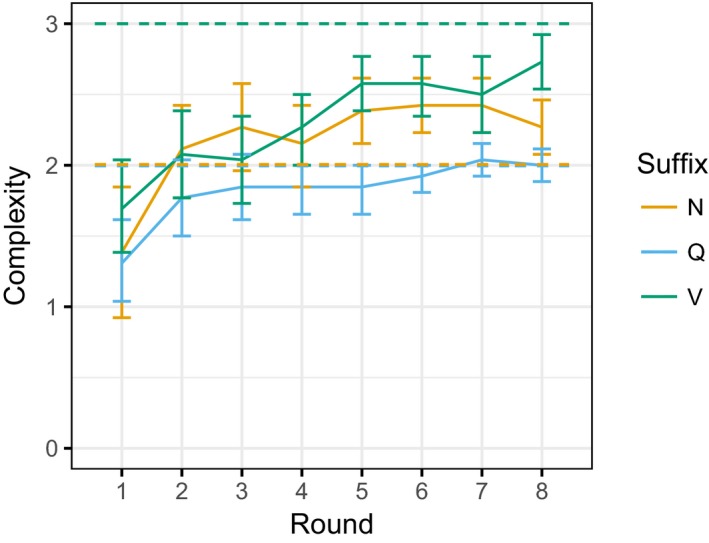
Average complexity for each suffix type (solid lines) and corresponding complexity for the target language (dashed lines). Complexity increases with training for all suffix types, although it does not always converge to the complexity of the target language. Error bars are 95% confidence intervals.

The resulting complexity values are plotted in Fig. 2. These complexity scores were submitted to an ordinal regression (using the ordinal package in R, Christensen, 2015) with round as a fixed effect and by‐participant and by‐suffix‐type random intercepts and random slopes for round (round in all cases being re‐valued so that the model intercept reflects complexity at Round 1). This model indicated a significant effect of round (*b *=* *0.294, *SE* = 0.060, *z* = 4.883, *p* *<* .001), reflecting the fact that all suffix types increase in the complexity of their conditioning with further training. As can be seen from the figure, however, only Q suffixes on average converge to the complexity of the target language: N suffixes tend to be a little more complex than the target language (reflecting additional irregularities, as seen in the Round 2 data in Table 2), whereas V suffixes tend to be somewhat less complex.

### 2.3 Experiment 1 discussion

The morphological systems of the languages produced by the participants after two rounds of training are simpler than those produced after eight rounds of training: the choice of suffix is conditioned in more complex ways at Round 8 than at Round 2 for all suffixes (see Fig. 2). This therefore confirms that learners produce quantifiably simpler morphological systems in the early stages of their acquisition, when they have received less input data. With sufficient training, our learners do eventually manage to acquire a close approximation of the target language, mastering its various complexities. In a more naturalistic language learning setting, with a more challenging target language to acquire, adult learners may never reach native‐like competence (Selinker, 1972). However, for our purposes, this difference between performance early and late in learning provides a model for differences between non‐native and native competence: Early learners in our experiment mirror the tendency of non‐natives to produce simplified systems, whereas late learners largely acquire the full complexity of the system in the same way as native speakers in the natural language case. Perhaps surprisingly, given the literature on adult learning of inflectional morphology, though the languages produced at Round 2 are morphologically simpler than the target language, this was not primarily due to the participants dropping the suffixes altogether. Only 5% of the Round 2 suffixes were zero‐marked, and so the simplified systems were more typically characterized by use of a smaller number of suffixes, conditioned a lower number of semantic features. Simplification here more typically involved the overgeneralization of one of the inflected forms of the target language.

Experiment 1 therefore shows that, in the early stages of learning, adult learners reduce morphological complexity. However, as discussed above, this is not in itself an explanation for why languages with larger proportions of non‐native speakers have simpler morphology: Unless the complexity of the language as a whole were defined as some average measure of the complexity of the individual languages of all of its speakers, simplification by individuals can only explain how a subset of a population could acquire and produce a simpler version of the language. A mechanism by which the simplifications of these non‐native learners can affect the language at the level of the population remains to be identified. In Experiment 2, we explore whether learners exposed to a mix of simplified and complex linguistic input will preferentially acquire the simpler variants, therefore offering an explanation for how such variants might spread through a population.

## 3 Experiment 2: Mixed linguistic input data and the spread of simplifications

The languages produced by our Experiment 1 participants at Round 2 provide a naturalistic set of morphological systems which are simpler than the complex target language. By Round 8, however, the languages our participants produced were typically very similar if not identical to those of the target language (21 of the 26 participants having a suffix success score greater than 0.90) and exhibit a comparable degree of morphological complexity. In Experiment 2, we use these languages produced as outputs in Experiment 1 as inputs for a second set of learners (using a subset of Experiment 1 participants who were particularly competent at learning the stem forms of the target language, see below). For our new Experiment 2 learners, we manipulate two aspects of the composition of their input data, according to a 2 × 2 between‐subjects design: its complexity and the number of Experiment 1 participants it is drawn from, which, though we recognize that input variability may not be the only relevant difference between language learning in a relatively large or small population, we take here as an experimental proxy for population size. This experiment allows us to test whether the simplifications learners produce in Experiment 1 will spread through subsequent learning in four different types of population. We investigate both the effects of the proportion of simplified data produced by adult learners in a learner's linguistic input—the proposed direct influence of adult learners on linguistic complexity—and the absolute number of speakers who provide the input, to allow for the possibility that population size itself has some influence on the input which may indirectly influence complexity (Lupyan & Dale, 2010).

Participants either receive *Complex* input, in which their training data is entirely composed of Round 8 output of participants from Experiment 1 (i.e., is uniformly morphologically complex), or *Mixed* input, in which they receive a combination of data from the relatively simple Round 2 output from Experiment 1 and the complex Round 8 output. This manipulation allows us to investigate whether learners exposed to a mix of naturalistically varying simplified and complex input preferentially acquire simplified morphological systems—if so, the simplifications produced by non‐native speakers would be predicted to spread.

To manipulate population size, we simply manipulated whether participants received input from data generated by two Experiment 1 participants (our *Small* condition) or data from a group of eight Experiment 1 participants (our *Large* condition). This manipulation allows us to test whether absolute number of speakers has an impact on learner behavior; in combination with the manipulation of input complexity, it allows us to check whether it is the proportion of simplified input sources, or their absolute number, which influences the way in which simplified forms are adopted (or fail to be adopted) by new learners. If the proportion of simplified input is the driver of simplification at the level of the language, then the existence of simplified input would result in the acquisition of simpler morphological systems, but group size would have no effect.

### 3.1 Participants

Forty‐eight native English speakers (27 female, 21 male; aged between 19 and 40, *M *= 23.2) were recruited using the Student and Graduate Employment (SAGE) database of the Careers Service at the University of Edinburgh. Each was compensated £7. Twelve participants were randomly assigned to each of the four conditions described below.

#### 3.1.1 The target language

As in Experiment 1, participants were trained on an artificial language which provides descriptions for scenes involving animals performing movements, the same scenes as in Experiment 1. Rather than being exposed to an experimenter‐designed language, during training our Experiment 2 participants were exposed to a set of descriptions obtained from our Experiment 1 participants, with the exact composition of that set of descriptions depending on the manipulations of input complexity and population size.

The Experiment 1 participants who had best acquired the stems in Round 2 were identified; specifically, those whose stem productions *exactly* matched the stems of the target language for at least 90% of the labels. 12 participants met this criterion, and had an average Round 2 stem success score of 0.99. The Round 2 stem and suffix productions of these participants provides a set of simplified input data which (as discussed above) contains morphological simplifications analogous to those produced by non‐native, adult learners of natural languages. The Round 8 data for these same 12 participants, with average stem success of 1.00 and average suffix success of 0.98, provides a set of complex input data—again, as discussed above, while this is the product of adult learning here, the fact that these languages accurately reflect the complex morphology of the Experiment 1 target language allows us to treat this complex data as a proxy for the output of competent, native speakers of a morphologically complex natural language, with only some minor variations in production amongst speakers. We used a subset of our Experiment 1 participants in order to avoid substantial variation in the stems the learners received as input, particularly for the Round 2 data. This subset of 12 Experiment 1 participants is generally representative of the full set of Experiment 1 participants; see Supplemental Information S3 for details.

The way in which the data from these Experiment 1 participants was combined to provide input for our Experiment 2 participants depended on condition. Our 2 × 2 manipulation of population size and input complexity leads to four different conditions: *Small‐Complex*,* Large‐Complex*,* Small‐Mixed*, and *Large‐Mixed*. In the Small‐Complex condition, two participants were selected from our pool of 12 Experiment 1 participants, and the descriptions they produced in Round 8 were used as training data. In the Large‐Complex condition, eight participants were selected from our Experiment 1 pool, and their Round 8 descriptions were used as training data. In the Small‐Mixed condition, two participants were again selected at random from our Experiment 1 pool; we took the (relatively simple) Round 2 data from one of these participants and the (relatively complex) Round 8 data from the other, and combined these to form a set of training data. Finally, in the Large‐Mixed condition, we took eight participants from our Experiment 1 pool, and used the Round 2 data from four of them and the Round 8 data from the other four to form a new set of training data.

#### 3.1.2 Procedure

The general experimental design closely follows that of Experiment 1. Participants were again required to learn a language which described the stimulus set of Supplemental Information S1 and again received eight rounds of training and testing following the same procedure.

As in Experiment 1, in each *training phase,* nine scenes were randomly selected for input. For a given participant, these nine scenes were then equally divided across the Experiment 1 participants providing the training data for that participant, and the description produced for that scene by the relevant Experiment 1 participant was used as the training description: in Small conditions, at each training phase the learner would therefore see nine descriptions, five produced by one Experiment 1 participant contributing to their input, and four provided by the other; in Large conditions, the nine descriptions would be produced by the eight Experiment 1 participants contributing to the learner's input, with one Experiment 1 participant contributing two descriptions and the rest contributing one each. The description provided by the chosen participant in Experiment 1 was paired with the corresponding scene, and these training items were presented as in Experiment 1. The participants were not informed that the input came from multiple participants at any point, and there was no overt marking of “speaker” identity; in other words, the only cue to the learner that their input came from multiple sources was highly indirect, and could only be inferred via variation in the forms used. We return to this point in the discussion. As the images and participants providing input descriptions were randomly selected for each training phase, a participant could potentially see the same image with a different label from one round to the next.

### 3.2 Analysis and results

Stem and suffix acquisition, and the complexity of the participant productions, are assessed using the same measures as in Experiment 1. The key difference is that we are now interested in a comparison of the final (Round 8) productions of each language across the conditions, as opposed to looking at how mastery of the input language changes over rounds. Details of a meaning‐independent measure, the entropy of the suffixes, are provided in Supplemental Information S4.

#### 3.2.1 Complexity of conditioning

**Figure 3:**
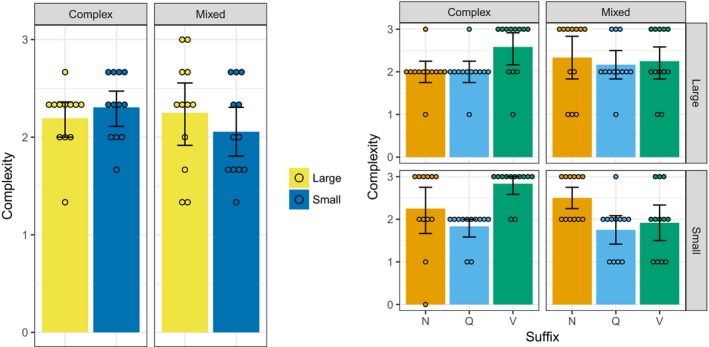
Complexity by condition and word type. The left plot shows mean complexity averaging over the three suffix types; the right‐hand panel shows complexity broken down by suffix. There is no evidence of a condition‐dependent difference in complexity at Round 8. Error bars are 95% confidence intervals. Points illustrate data from individual participants.

As for Experiment 1, we found the best‐fitting (by AIC) multinomial model predicting suffix choice for each participant's data for each suffix type, here focussing on their Round 8 productions. These complexity scores are plotted in Fig. 3. Again, while there seems to be some variability in the complexity of the suffixes, there is little evidence of any systematic differences based on population size or input composition. This impression is confirmed by an ordinal regression predicting complexity from population size, input composition, and their interaction (with the same random effects structure as the entropy analysis above: by‐participant random intercepts; by‐suffix‐type random intercepts and random slopes for population size, input composition and their interaction). The fitted model shows no significant effects (lowest p observed for the interaction between population size and input composition: *b *=* *−1.365, *SE* = 0.964, *z* =* *−1.416, *p* = .157).

#### 3.2.2 Experiment 2 discussion

Experiment 2 therefore offers no support for the hypothesis that receiving input consisting of a mix of simplified and complex versions of a morphologically complex target language is enough to engender simplification in a second generation of learners. As such, it fails to support one account by which individual adult learners simplify a language solely by their simplifications being introduced into the linguistic input for subsequent learners.

In the Mixed conditions, the participants received a mix of simple and more complex morphological systems. Compared to the participants in the Complex conditions, who were only exposed to complex morphological data, it is therefore rather surprising that they did not acquire simpler systems. Counterintuitively, we find that the mixing process did not necessarily reduce complexity in a participant's input; mixing multiple simplified versions of the language does not necessarily yield a simplified set of input, because our Experiment 1 participants simplified in different ways. For example, while the quantifier suffixes for our example participant in Table 2 at Round 2 were entirely conditioned on Number with*‐a* for singular and*‐ak* for plural, another participant's Round 2 quantifiers, though also simpler than the target language in being entirely conditioned on Number, had*‐u* for singular and*‐uk* for plural. Combining such idiosyncratically simplified systems yields a pooled set of data which is highly variable and therefore, according to our measures of complexity, itself highly complex. See Supplemental Information S5 for details.

Experiment 2 participants were therefore exposed to complex input and produced complex output, regardless of where the complexity of their input came from (either through consistently reproduced versions of the complex target language from Experiment 1 or complexity generated by mixing multiple simplified systems). Similar effects of mixing are reported in Smith et al. (2017) in a computational model and human experiment exploring the transmission of unpredictably variable linguistic systems: While individual learners tend to condition linguistic variants on aspects of the linguistic context, there is substantial inter‐individual variation in which variants are conditioned on which contexts; consequently, as we see here, mixing the output of multiple such individuals masks this individual‐level simplification of the language.

One obvious factor which might have influenced our results is that our Experiment 2 learners were not able to track which speakers generating their input were responsible for which descriptions. For instance, providing information on speaker identity (e.g., by tagging each description with an avatar corresponding to the individual who produced it) would in principle allow learners in Mixed conditions to identify that there was a consensus of a morphologically complex language used by half of the individuals providing their training data, then a more individually variable but simpler set of languages produced by multiple other individuals. Smith et al. (2017) provide models showing that, at least for the case of variation‐learning, such speaker‐based conditioning can in some circumstances attenuate the effects of mixing; for instance, if an individual identifies with a single input source (say based on shared social characteristics) and then bases his or her own output on that individual, effects of mixing are entirely removed.

There are two caveats in order here, however. Firstly, social identity might allow learners to account for the variability they perceive in their input, but doing so would require them to be able to track how speaker identity predicts lingustic complexity, that is, track which speaker(s) produce labels from comparatively simple and complex systems.

This is itself is likely to be challenging: while a wealth of sociolinguistic research shows that linguistic variation is socially conditioned, there is a debate about whether social conditioning is easy or hard to acquire, relative to linguistic conditioning factors (see, e.g., reviews in Samara, Smith, Brown, & Wonnacott, 2017; Shin, 2016). Furthermore, a recent artificial language learning study using a variation‐learning paradigm (Samara et al., 2017) found that, while learners (both adults and children) are capable of learning systems of socially conditioned variation, doing so is relatively difficult compared to, for example, learning linguistically conditioned variation. In the same vein, in their experimental study Smith et al. (2017) manipulated the presence or absence of speaker identity information while mixing input from multiple speakers, and found that it made no difference to the mixing effect, again suggesting that learners can at least sometimes fail to exploit social cues which would otherwise allow them to account for variability in their input. Providing information on speaker identity therefore does not guarantee that participants will be able to use it to deal with variability in their input, and the requirement to track speaker identity may in itself add to the burden on learners.

Secondly, using speaker identity to account for the variability in their input does not guarantee that in such circumstances learners will preferentially acquire a simpler morphological system when presented with a consensus of a complex system and multiple idiosyncratic simpler systems. Indeed, the most obvious prediction here is that providing speaker identity would further reduce differences between conditions: Given the prevalence of conformity effects in social learning, we might expect that learners provided with cues to speaker identity would preferentially acquire the morphological system shared across most input speakers, that is, the complex language. If so, there would be even less reason to expect idiosyncratic simplifications introduced by individuals to spread through populations.

Of course, it is also possible that our failure to show that simplifications spread might be an artifact of some detail of our experiment; for example, if there was greater consensus among our Round 2 learners about how to simplify (e.g., if most early learners opted for zero‐marking), then we may have seen simpler languages winning out in mixed input conditions. Alternatively, it may be that a simplification effect of mixing the different input types may only become apparent with multiple generations of learning (in line with, e.g., Kirby, Dowman, & Griffiths, 2007), although Smith et al. (2017) show that transmission is not guaranteed to overcome the effects of mixing.

These concerns could be addressed in future experiments, but this study has at the very least demonstrated that an influence of individual‐level simplification on group‐level language complexity cannot be taken for granted. In assessing the acquisition of informationally redundant morphology, we have examined one type of language feature specifically proposed to simplify under pressure of adult learning (Lupyan & Dale, 2010), and we found that simplifications introduced early in learning do not preferentially spread. In Experiment 3, we therefore consider the effect of language use and specifically the consequences of interaction between individuals differing in the complexity of their languages, exploring accommodation during interaction as an alternative mechanism by which simplified forms might spread through a population.

## 4 Experiment 3: Learner simplifications and speaker accommodation

Speakers typically accommodate their interlocutors, making both linguistic and extralinguistic adjustments which facilitate interaction (e.g., Giles, Coupland, & Coupland, 1991; Reitter & Moore, 2014), and pursue socially affective goals (van Baaren, Holland, Steenaert, & van Knippenberg, 2003). This leads to both parties producing similar forms or constructions at the phonetic (Giles et al., 1991), lexical (Brennan & Clark, 1996; Garrod & Anderson, 1987), semantic (Garrod & Anderson, 1987; Garrod & Clark, 1993), and syntactic (Bock, 1986; Gries, 2005) levels. Alignment effects have also been demonstrated in children (e.g., Branigan, Tosi, & Gillespie‐Smith, 2016) and in artificial language learning experiments (Fehér et al., 2016).

Alignment does not necessarily involve both speakers adjusting their speech to facilitate the interaction to the same extent. Where a proficient speaker of a language is communicating with a less proficient speaker, such as a child or a non‐native speaker, or where a more proficient non‐native speaker interacts with a less proficient non‐native, there may be *asymmetric alignment* (Fehér et al., unpublished data), where the proficient speaker makes linguistic adjustments to a greater extent. This is particularly evident in the case of infant‐directed speech and foreigner‐directed speech (Uther, Knoll, & Burnham, 2007).

Foreigner‐directed speech is of particular relevance to our questions regarding the role of adult learning in language simplification. By comparison to infant‐directed speech, foreigner‐directed speech is far less extensively researched (Uther et al., 2007), and what research there is focuses heavily on the English language and Western culture. Wesche (1994) summarizes its main characteristics. Speech rate is lower, with exaggerated intonation and stress on topic nouns, and with more frequent and longer pauses. This leads to more careful (hyper)articulation of underlying vowels and consonant clusters (see Xu, Burnham, Kitamura, & Vollmer‐Conna, 2013, for a comparison of vowel hyperarticulation effects in different types of directed speech), and avoidance of contractions, which makes word boundaries more clearly defined. Utterances are shorter and syntactically simpler. They are grammatically well‐formed, except in the case of fragments used to repeat or elicit information for didactic purposes. Canonical word order is frequently used. Optional grammatical forms are retained and the present tense used more often. Vocabulary is less varied, with a greater use of copulas, and full noun phrases with proper nouns preferred over pronouns. There is also more repetition, comprehension checks, clarification requests, restatements, expansion of hearer utterances, and closed questions (Long, 1981; Wesche, 1994). These various features are more exaggerated when the hearer is less proficient.

In addition to these observational findings, there is also some experimental evidence on how non‐nativeness affects accommodation during interaction. Chun, Barrow, and Kaan (2016) show that perceived linguistic knowledge can influence alignment in natural language. They found that native speakers of American English were more likely to use a preposition‐object construction over a double‐object when primed to do so by either a native speaker of Indian English or a non‐native (Korean) speaker, rather than by a native speaker of American English: Alignment was greater when the participants were exposed to the accents which differed more greatly to their own. In an experimental investigation of asymmetric alignment, Fehér et al. (unpublished data) trained pairs of participants on miniature languages which featured an optional grammatical marker, and then had them use that language to communicate with each other. One participant in each pair was trained on a relatively complex language in which the marker was sometimes used and sometimes not; their partner was trained on a simpler, categorical system where the marker was used obligatorily. Results were consistent with the asymmetric alignment hypothesis: variably trained participants accommodated to their categorically trained partners, who did not change their behavior.

How could these types of alignment phenomena influence language complexity? If more proficient speakers accommodate to comparatively less competent speakers by simplifying their language, then these simplifications will form part of the ambient environment from which child learners receive their linguistic input. The spread of these simplifications will still depend on the treatment of input variability for subsequent learners, but the frequency of the simplifications will at least have increased in that input. There is also the possibility that, through sufficient exposure to the simpler language of non‐native speakers and experience of using simplified, foreigner‐directed speech, native speakers will incorporate simplifications into their own language, and so these simplifications may spread through horizontal transmission, further increasing the frequency of simplified forms. Dale and Lupyan (2012) provide some evidence for such effects of interaction with non‐native speakers, finding that adult native English speakers who had had a greater amount of contact with non‐native speakers showed a greater preference for regularized variants of irregular past tense verbs (e.g., “speeded” compared to “sped”).

In Experiment 3, we therefore explore this potential mechanism by assessing how a language may change as a result of a proficient speaker of a complex language (a *Complex speaker)* communicating with a less proficient speaker (a *Simple speaker)*. Complex speakers are participants trained to criterion on a complex artificial language (exhibiting both regular and irregular forms of a “verb”). Simple speakers are trained on a simpler variant of this language in which they are only exposed to the regular components. The expectation is that this second group of speakers will then acquire a fully regular version of the target language in which they produce regular forms for both the regular and irregular items. As discussed in the Introduction, such acquisition difficulties with irregularities (Trudgill, 2011; Wray & Grace, 2007) and invariance of form in production (Klein & Perdue, 1997) is symptomatic of the adult learners (though again note Cuskley et al., 2015). We then compare two different cases of dyadic interaction: *Complex dyads* involving two Complex speakers, and *Mixed dyads*, involving a Complex speaker and a Simple speaker. In the Complex dyads, no change in the language is predicted as a result of the interaction (in this respect, this is a control condition). In the Mixed dyads, it could be the case that participants trained on the version of the language lacking irregulars simply acquire those forms from their partner, resulting in a net gain in complexity due to interaction; alternatively, a decrease in the complexity of the language of the speaker trained on the complex language, to accommodate the less proficient speaker, would demonstrate how simplifications of the type proposed to arise from adult learning could spread to other individuals.

### 4.1 Methods

Our method here shares some features with Experiments 1–2, in that we train participants on an artificial language exhibiting some irregularity, although here we use a simpler target language.^5^ In order to study the effects of interaction, after participants are trained on the language (or a subset thereof; see below), they play a simple director‐matcher game with another participant, taking turns to describe scenes to each other using the artificial language. Finally, we provide a post‐interaction recall test (carried out as individuals, rather than in interaction with their partner, as in Fehér et al., 2016), to assess the extent to which any simplifications or increases in complexity occurring during interaction outlast the course of that interaction.

#### 4.1.1 Participants

Here, 60 native English speakers (43 female, 17 male; aged between 18 and 34, *M = *19.8) were recruited at the University of Stirling. Each was compensated either two Psychology course tokens and £3, or £7. Twenty participants were assigned to the Complex dyads condition, yielding 10 dyads in that condition; the remaining 40 participants were assigned to the Mixed dyads condition, yielding 20 dyads in that condition. Data for six further participants (3 dyads) was also collected, but it is not included in this analysis as one or both of the participants in each dyad failed to meet the training criterion in the maximum 10 rounds of training and testing.

#### 4.1.2 The target language

We reduced the size of the target language by reducing the set of scenes to be labeled to nine items, featuring all combinations of three animals (duck, dog, and crocodile; the bird from Experiments 1–2 was replaced by a dog so as to be more obviously different to the duck) and three different movements (straight, bouncing, and looping). The set of images is given in Supplemental Information S6.

Morphological complexity was represented by a combination of regular and irregular verbs. The target language was constructed using three transparent pseudo‐English nouns labeling the three animals: *kwako*,* grolo*, and *snapo*. A given image's description was then made up of one of these nouns and a verb. We generated a set of five artificial words to use as verbs (*jing*,* rald*,* nunj*,* ferb*, and *yath*). For each dyad, we selected three of these forms at random and assigned them to the three movements, to function as the regular verbs. The other two forms were used as irregular verbs and replaced a randomly selected two of the regular verbs for a particular noun–verb combination, with the stipulation that there would be at most one irregular verb for each animal, and at most one for each movement. These target languages therefore exhibit condition variation; while the variation is lexically conditioned, such a pattern of conditioned variation is analogous to that which occurs in, for example, gender systems or irregularities in past tense marking. An example target language is given in Table 4.

**Table 4:** Example target language

Scene	Description
duck straight	kwako jing
dog straight	grolo jing
crocodile straight	snapo jing
duck bounce	kwako yath
dog bounce	grolo *nunj*
crocodile bounce	snapo yath
duck loop	kwako *rald*
dog loop	grolo ferb
crocodile loop	snapo ferb

Note

The two irregular verbs are highlighted in italics.

#### 4.1.3 Procedure

The experiment had four stages: noun training, sentence training, interaction, and a final individual testing stage.

In the *noun training stage,* participants were trained to criterion on the nouns of the target language. Noun training consisted of three phases:

- The participants were first exposed to the three nouns. As for the training trials in Experiments 1 and 2, each image was presented for 1 second in isolation, before the label was also presented as text for a further 5 s. The participants then had to retype the label from memory. No feedback was given as to the accuracy of the retyping. Each animal–label pair was presented once.
- After this initial pass over the set of nouns, the participants then alternated between two types of training trial (as in Culbertson et al., 2012), both of which exposed participants to animal–label pairs from the target language. In *retyping trials*, they were given the animal–label pair and required to retype the label, as above. In *referent‐selection trials*, they were presented with a label alongside a selection array of all three animals, and were required to select the correct animal. Feedback was given as to whether their choice was correct or incorrect, and then the incorrect images were removed so that the correct pairing was made explicit. Both trial types therefore feature an unambiguous exposure to the correct animal–label pairing but differ in whether the participant needs to recall the label or its referent.
- Finally, the participants were tested on their ability to label all three animals. No feedback was given. If both participants reproduced the nouns with 100% accuracy, the training moved on to sentence training. If one or both of the participants made any errors in the final test, alternating training steps (ii, above) and testing (iii) were repeated (for both participants) until the nouns had been learned, or until the pair reached 10 training‐test cycles, in which case they progressed to the next stage of the experiment but the data from that dyad was excluded from the analysis.

Once the dyads had completed noun training they progressed to *sentence training*. Sentence training worked in the same way as noun training, but involved training participants on image–label pairings rather than animal–noun pairings (where images feature an animal with an indication of movement and labels feature both a noun and a verb): First, there was an initial retyping pass, followed by alternating training and testing phases until both participants had reached criterion. The input participants received during sentence training depended both on the experimental condition (Complex dyads or Mixed dyads), and the role they were allocated within that dyad (Complex speaker or Simple speaker). In Complex dyads, both participants were trained on the full target language. In Mixed dyads, one participant (the Complex speaker) was trained on the full language, while the other (the Simple speaker) only received the seven regular image–label pairings during training. In the Table 4 language, for example, the pairings for “dog bounce” and “duck loop” would be withheld for the Simple speaker. For Complex speakers, the criterion to progress was 100% accurate reproduction of all verbs of the target language. For Simple speakers, this was 100% accurate reproduction of the regular verbs; these participants were not required to describe the two images which they did not encounter in training. Again, both members of the pair repeated the training‐test loop until they both reached criterion.

The training stage was followed by the *interaction stage*. In interaction, participants in a dyad took turns directing and matching. When asked to direct, the participant was shown a scene and required to produce a description. When asked to match, the participant received the director's description and was required to select the corresponding scene. All nine scenes were displayed on the matcher's screen in a random order. After the matcher made their selection, both the director and the matcher received feedback: a green success screen or a red failure screen was displayed, along with a cumulative success score. Participants were instructed to achieve as high a communicative success score as possible. In each round of interaction each participant directed all nine scenes in a random order; the interaction phase lasted for three rounds total.

In the *final testing phase*, the participants were again required to produce descriptions for each of the nine scenes. This post‐interaction individual test allows us to investigate if any accommodation behavior exhibited by a participant during the interaction was retained. This was presented to the participants as a “final test,” and they were simply instructed to “label each image.”

The experiment was written and run in PsychoPy v1.83 (Peirce, 2007).

### 4.2 Analysis and results

#### 4.2.1 Acquisition of the training data

The training of the nouns in isolation was predictably trivial. Fifty‐three of the 60 participants reached criterion in the first test; the remaining 7 took 2 tests. Training on full descriptions took longer: the 40 complex speakers took between 2 and 10 training‐test cycles to acquire the full language; average 4.9 cycles. The 20 simple speakers trained only on the regulars took between 1 and 9 training‐test cycles to acquire the restricted language; average 2.1 cycles.

#### 4.2.2 Communicative success

**Figure 4:**
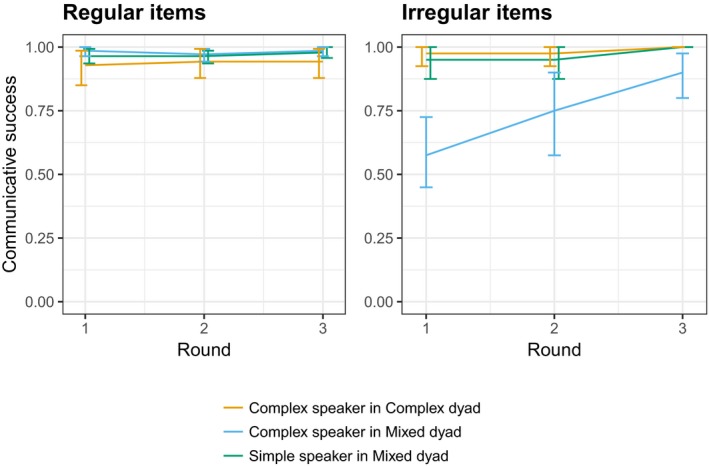
Proportion of successfully communicated test items by round and condition for the regular items (left) and the irregular items (right). Within the Complex speaker productions, communicative success is greater in the Complex dyads than in the Mixed dyads: Complex speaker labels were more accurately matched by other Complex speakers than by Simple speakers. Error bars are 95% confident intervals.

Average communicative success, where a directed label was correctly matched, across all interactions was 95%: 96% for regular items in the full language and 90% for the irregular. The average communicative success scores by round, speaker, dyad type, and test item type (regular or irregular in the full language) are shown in Fig. 4. On average across all rounds, communicative success for irregular items was 98% for Complex speakers in Complex dyads, 74% for Complex speakers in Mixed dyads, and 97% for Simple speakers in Mixed dyads.

We compare the effect of dyad type on communicative success within the Complex speakers, using a logit mixed model with dyad type (Complex or Mixed), round (revalued such that the model intercept reflects the success score at Round 1), and their interaction as fixed effects, with by‐participant random intercepts and by‐participant random slopes for round (participant identity nested within dyad). Complex speaker in Complex dyads was taken as the baseline. The model indicated a significant effect of interaction type (*b *=* *−3.456, *SE* = 1.048, *z* =* *−3.298, *p* < .001), but no effect of round (*b *=* *0.860, *SE* = 1.097, *z* = 0.783, *p* = .433), or their interaction (*b *=* *0.190, *SE* = 1.093, *z* = 0.174, *p* = .862); unsurprisingly, given the differences in the training for the matchers in each case, the labels produced by the Complex speakers in Mixed dyads were less successfully matched than those of the Complex speakers in Complex dyads. Note that lack of an interaction effect here is likely due to the majority of the items being regular, and the analysis making no distinction between the regular and irregular items.

#### 4.2.3 Regularization in interaction

**Figure 5:**
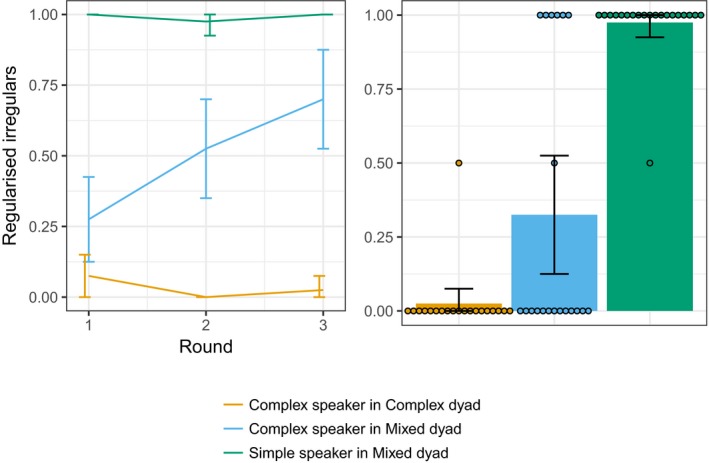
Proportion of regularized irregulars in interaction (left) and in postinteraction individual testing (right). Complex speakers in Mixed dyads produced more regularized irregulars during interaction than Complex speakers in Complex dyads, particularly at later rounds. In the post‐interaction recall test, Complex speakers in Mixed dyads also produced more regularized forms than Complex speakers in Complex dyads; to an extent, the regularization persists post‐interaction. Error bars are 95% confidence intervals. Points illustrate data from individual participants.

Over all three rounds of interaction, Complex speakers in Complex dyads on average regularized the irregular verbs in 3% of trials involving an irregular (4 of 120 productions). In the language in Table 4, for example, this could be by using the regularized description *kwako ferb* to communicate the “duck loop” image. By contrast, Complex speakers in Mixed dyads regularized 50% of the time (60 of 120). Simple speakers in Mixed dyads (who were not taught the irregulars in training) produced regularized variants of the irregulars 99% of the time (119 of 120). The proportion of regularized verbs produced by speaker and dyad type is shown in Fig. 5.

Within the Complex speakers, we investigate the effect of dyad type on regularization of the irregular items. Logit mixed effects analysis again included dyad type (Complex or Mixed), round (again with the model intercept reflecting Round 1), and their interaction as fixed effects. Complex dyad was taken as the baseline condition, and we included by‐participant random intercepts and by‐participant random slopes for round (participant identity nested within dyad). There was no significant effects of dyad type (*b *=* *2.148, *SE* = 1.184, *z* = 1.815, *p* = .070), and no effect of round for the baseline Complex dyad condition (*b *=* *−2.208, *SE* = 1.484, *z* =* *−1.488, *p* = .137); however, the interaction of round and dyad type was significant (*b *=* *4.200, *SE* = 1.724, *z* = 2.437, *p* = .015), indicating that Complex speakers in the Mixed dyad condition increased their use of regularized forms over rounds.

Complex speakers in Mixed interaction dyads were therefore more likely to regularize the irregular verbs they acquired in training than those in Complex interaction. They were also more likely to do this in later rounds, after they had had a greater amount of interaction with a Simple speaker. This regularization clearly aided communication, with Simple speakers correctly matching (i.e., identifying the intended referent of) 98% (59 of 60) of the regularized forms produced by Complex speakers, compared to only 50% (30 of 60) of the irregular forms. Complex speakers also clearly understood the regularized forms produced by the Simple speakers, correctly matching 97% (116 of 119). The single irregular form produced by a Simple speaker was incorrectly matched. In a logit fit model for the irregular items produced by the Complex speakers in Mixed dyads, with communicative success as dependent variable, regularization as fixed effect, and random intercepts for participant identity and round, there was a significant effect of regularization (*b *=* *4.146, *SE* = 1.069, *z* = 3.879, *p* < .001).

Regularization by Complex speakers in Mixed dyads therefore improved communication, so we examine what elicited it. These participants used a regularized form for an irregular item in at least 1 of the 3 rounds in 29 out of the 40 cases (there were 2 irregular items to label per Complex speaker in a dyad and 20 dyads). In 27 of these 29 cases, the first time they produced a regularized form followed at least one production of that exact form by the Simple speaker (in 11 of these 27, the Complex speaker had also produced the irregular which was at least once incorrectly matched and never correctly matched); in the remaining two cases, the Complex speaker produced the regularized form without first encountering it from the Simple speaker, but in each case they had already encountered the regularized form of the other irregular item in the Simple speakers productions, so had had some exposure of the Simple speaker producing a simpler variant of the language. When Complex speakers regularized, therefore, it appeared to be in response to the regular forms being produced by their partners, possibly with an additional influence in some cases of having produced irregular forms which lead to unsuccessful communication.

Once a Complex speaker produced a regularized form for an irregular item, they typically continued to do so. There were 22 cases where a Complex speaker produced a regularized form for the first time and then had another opportunity to label the irregular item (i.e., their first regularized production was in Round 1 or 2, not the final Round 3). In 21 of these 22 cases, Complex speakers stuck with the regularized form after producing it once; only in one case did we see a Complex speaker reverting to an irregular form.

#### 4.2.4 Regularization in post‐interaction individual testing

The proportion of regularized irregulars produced in post‐interaction individual testing is also shown in Fig. 5. 7 of the 20 Complex speakers in Mixed dyads produced regularized forms of the irregular verbs for at least one irregular verb during the post‐interaction individual testing phase, compared to only 1 of the 20 Complex speakers in Complex dyads, a significant effect of dyad type (χ^2^(1) = 5.626, *p* = .018).^6^ Of the Simple speakers in Mixed dyads, only 1 of 20 participants did not produce regularized forms for both irregular items, producing one irregular label which they encountered from their Complex speaker partner during interaction.

### 4.3 Experiment 3 discussion

Complex speakers simplified the target language in interaction with Simple speakers, by regularizing irregular forms and hence eliminating the conditioned variation. Such simplification was not necessary in the Complex dyads, as both participants in the dyad had learned the same full language, including the irregular verbs. In demonstrating that individuals simplify their language to aid communication with interlocutors who have acquired a simpler version of the target language, this experiment demonstrates how native speaker accommodation to adult learners may lead to language simplification. Firstly, in replacing irregulars with regularized forms to match those of their interlocutors, our Complex speakers have simplified the language at the level of the interaction itself. Secondly, more proficient speakers’ use of simplified forms will increase their frequency in the linguistic input of subsequent learners. Unlike the idiosyncratic simplifications of Experiment 2, these simplifications will also be more standardized, in that the same simplifications are likely to be used by both the comparatively proficient and the less proficient non‐native speakers, in the case where the interaction is between two non‐native speakers, or the accommodating native speaker and the accommodated adult learner, where foreigner‐directed speech is involved. The simplifications are then more likely to be transmitted, and so accommodation may be a key linking mechanism by which the simplifications of adult learners spread.

After simplifying their language in interaction, some of the Complex speakers in Mixed dyads retained simplifications in individual testing. Of course, this retention of simplified forms may not be as striking in a more naturalistic setting, and further work is also necessary to determine how long such simplifications are likely to be retained and produced. It may be, for example, that a more proficient speaker may only continue to retain a given simplification with repeated interaction with less proficient speakers. Whether or not a proficient speaker produces a previously encountered simplification may also depend on previous interactions with their current interlocutor, or their perceptions of their interlocutor's proficiency. They may, for example, be less likely to produce a simplification when they have not heard that speaker produce that simplification themselves, when they have not had many interactions with that speaker before, or where that speaker is (or perhaps is not) a relatively new learner of the language. Even if a native speaker retains simplifications for a period after interaction with an adult learner, however, we would predict that subsequent interaction with other native speakers would largely eliminate the effect. With larger amounts of interaction with adult learners, though, non‐native simplifications may still have a more lasting effect on the language of the native speaker.

While our conclusions would seem relatively uncontroversial for communication between non‐natives of differing competences—our experimental participants here are all “non‐native speakers” of the language after all—it worth noting some possible objections to the case where is it is *native* speakers of a language producing the simplified forms. Firstly, it could be argued that accommodation such as that observed in this experiment is unlikely to occur in naturalistic foreigner‐directed speech. Foreigner‐directed speech has simpler morphosyntax than normal speech, but this is reportedly characterized by features such as shorter utterances, a greater use of canonical word order and the present tense, and retention of optional grammatical forms (Uther et al., 2007; Wesche, 1994). From the limited research available on foreigner‐directed speech, therefore, utterances are grammatically well‐formed (Wesche, 1994), while we have observed Complex language participants produce verb forms which differ from those they acquired. There is an increasing amount of evidence that there are fundamental differences between how native speakers interact with non‐natives and how they interact with other natives, however. Lev‐Ari (2015) and Lev‐Ari, Ho, and Keysar (2018) have demonstrated how language processing can depend on whether a native speaker is communicating with a native or non‐native speaker, while, as previously noted, Dale and Lupyan (2012) have observed over‐regularization of verbs in English which may be a result of interaction with non‐native speakers. Finally, Wiese's (2009) work on the dialects spoken in urban areas with large migrant populations illustrates how native speakers may adopt a grammatically simpler variant of their first language. Given the limited research on foreigner‐directed speech (Uther et al., 2007), particularly that focussing on languages other than English and non‐Western culture, such accommodation may be a feature of foreigner‐directed speech more generally. This type of accommodation may also be largely confined to cases where the more complex, unregularized form of a lexical item is regularized in response to comprehension failure on the part of the non‐native speaker (as opposed to the native speaker accommodating solely in response to the simplified productions of their non‐native interlocutor). If this the case, accommodation may be less likely to result in regularized form for comparatively transparent items such as “women” and “forgot,” than for “stole” and “thought,” which are perhaps less likely to be understood by a non‐native speaker.

A second objection relates to the restricted input received by the experiment's Simple speakers, and whether such a lack of exposure to irregulars is typical of non‐native learning. Irregulars are typically high frequency, and so it is likely that an adult learner actually would be exposed to them in a more naturalistic setting. In the case of very high‐frequency irregulars, it is certainly possible that they are the more likely elements to be acquired by a learner with limited input. More generally, however, and particularly for lower‐frequency irregulars, we would expect our pattern of results to hold: learners with less input acquire simpler systems (as demonstrated in Experiment 1), and the more proficient speaker in a dyad simplifies their productions to increase communicative success. This could be confirmed in future experiments based on the methodology of Experiment 3, with larger languages and the restricted‐data participants being exposed to the entire target language (although still receiving less input overall compared to the Complex speakers).

## 5 General discussion

Taken together, these experiments do support the hypothesis that adult learning can reduce the complexity of a language, but they also highlight the need to test the mechanisms by which the simplifications arising from adult learning may influence complexity at the group level. Accommodation by more proficient speakers to the less proficient may be such a linking mechanism.

Experiment 1 demonstrates that learners, with reduced exposure to a target language, acquire simplified morphology. This is in line with previous empirical work (Clahsen et al., 2010; Gürel, 2000; Haznedar, 2006; Klein & Perdue, 1997; Papadopoulou et al., 2011; Parodi et al., 2004), and proposals that complex language features, such as informationally redundant morphological inflections, are challenging for adults to acquire (Kusters, 2003; Lupyan & Dale, 2010; McWhorter, 2007; Trudgill, 2011; Wray & Grace, 2007). Experiment 2 then mixes such simplifications with more complex, “native‐like,” language to investigate how the simplifications may spread through subsequent learning, investigating both the effects of the proportion of simplified data in a learner's linguistic input—the proposed direct influence of adult learning on linguistic complexity—and the absolute number of speakers who provide the input, to allow for population size itself having some influence on the input which may indirectly influence complexity (Lupyan & Dale, 2010). We found no condition‐dependent differences in the complexity of the acquired languages, and so no evidence to support a view that a mix of simplified and complex versions of a morphologically complex target language will result in simplifications propagating on its own. Instead, we see that mixing simplifications with complex language leads to linguistic input which is itself complex and variable, resulting in the complexity of the language being maintained. Experiment 3 suggests one mechanism by which the mixed input's variability and complexity could simultaneously be reduced: more proficient speakers of a complex language simplify their productions to facilitate communication with individuals who speak a simplified variant of the full language. In contrast to the simplifications in the input for the participants in Experiment 2, the effect of interaction is that the simplifications will be both more standardized and more widespread, and so more likely to spread to subsequent learners. These learners may be children, whose reduction of variable input (Culbertson & Newport, 2015; Culbertson et al., 2012; Hudson Kam, 2015; Hudson Kam & Newport, 2005, 2009; Wonnacott, 2011) will be more likely to include simplification if the input includes the accommodating speech of native speakers to non‐natives; or they may be adults, who will then be exposed to more standardized simplified variants of the target language, which has already adapted to the learning needs and preferences of other adult learners (the ones who produced the simplifications in the first place). Experiment 3 also demonstrates how interaction may lead to the spread of simpler systems through horizontal transmission, with some participants originally trained on the full language retaining the simplifications they encountered from their less‐proficient interlocutors in post‐interaction testing.

While these experiments do illustrate how adult simplifications could spread through learning and use to reduce the complexity of the language at group‐level, further investigation is required to assess the extent to which our findings hold in more naturalistic settings and for different features of linguistic complexity. We have observed simplifications arising from adult learning in Experiment 1, though Cuskley et al. (2015) have demonstrated that non‐native speakers do not necessarily produce simpler forms. Future work could explore under what circumstances, and for what features, adults predictably simplify linguistic systems. In Experiment 2 we also saw how mixing simplified and complex input will not necessarily lead to the acquisition of a simplified system by new learners; future studies could use our experimental method to test whether there are any situations in which simplifications will spread through learning alone. It may be, for example, that simplifications are propagated solely through learning when target languages featuring a different kind of complexity are involved. Future work is also necessary to determine the extent to which simplifications need to be standardized and high‐frequency in a learner's input in order for learners to reduce the complexity of the transmitted language. Additionally, as we argue in the Experiment 2 discussion, we would not anticipate a version of the experiment which included explicit marking of speaker identity to result in the propagation of simplifications, but we would still welcome further work which tested this experimentally.

It is also worth highlighting that in Experiment 2, we operationalized population size as the number of different speakers a learner received their input from. While we think it likely that a typical learner in a larger population will receive input from a greater number of speakers, speaker input variability may also be determined by other factors, such as the area the population is spread over. There may also be other relevant features of language learning in a larger group, such as the learner interacting with speakers with whom he or she shares less common ground. These may lead to mixed learning input having different effects on what the learner acquires, and so there may yet be some aspect of population size which leads to mixed learning input reducing the complexity of a language without additional mechanisms. Again, we would welcome further research in these areas.

Further investigation into the effects of accommodation between speakers of differing linguistic competence will also provide greater insights into the potential role of native speaker accommodation in language simplification. It would be useful to investigate the extent to which the more proficient speakers accommodate when exposed to differing degrees and types of simplification from their interlocutors. As with Experiments 1–2, future work could also investigate the extent to which our results here hold with different target languages. Though we would predict Complex speakers to accommodate to Simple speakers in languages with inflectional morphology, for example, confirmation of this would be welcome.

Experiment 3 also suggests that the Complex speakers produced simplifications in response to the regularizations produced by their Simple interlocutor, rather than in response to their own irregulars being misunderstood. A future study could also investigate the influence of comprehension failure more systematically, for instance exploring whether a Complex speaker would adopt regularized forms which they had not been training on when interacting with a speaker who consistently produced regularized forms, yet who also correctly interpreted the irregular forms as well.

An additional factor we have not considered is the interaction between two “non‐native” (i.e., simplifying) learners of similar proficiency. If such learners acquire (potentially different) simplified systems, similar interactive alignment effects may still serve to standardize the simplifications encountered by both native speaker interlocutors and subsequent learners exposed to their linguistic output, and this may also facilitate the propagation of simplifications. It would also be useful to know how representative our Complex speaker to Simple speaker accommodation is of naturalistic accommodation by native speakers to adult learners of a language, to build on evidence that contact with non‐native speakers may result in a more general preference for over‐regularization (Dale & Lupyan, 2012).

Finally, we note that there are alternative or additional potential explanations, besides those involving adult learning, for links between the number of speakers of a language and its complexity. Languages of larger groups may be structurally simpler due to their having more complex (non‐linguistic) culture, which has been shown to inversely correlate with the complexity of some language features (Perkins, 1992). Alternatively, their relative simplicity may be due to the situational contexts under which certain utterances are produced (see Winters, Kirby, & Smith, 2015, 2018; for how languages can adapt to their contextual niche), differences between what needs to be communicated in different sized groups (see Perfors & Navarro, 2014; for how languages adapt to the structure of environment they are used in), or as a consequence of the particular social network structure of larger populations (e.g., Reali, Chater, & Christiansen, 2014).

## 6 Conclusions

Over three experiments, we test the hypothesis that adult learning plays a crucial role in language simplification and drives the negative correlation between population size and linguistic complexity. While we do find that language use in the early stages of learning (our experimental proxy for adult learning) is characterized by simplification, we also find that the simplifications introduced in such circumstances are unlikely to spread through a population solely by subsequent learning, even if that population contains a large proportion of adult learners, or a large absolute number of adult learners. The simplifications that occur early in learning tend to be somewhat idiosyncratic, and consequently a subsequent generation of learners exposed to a mix of such idiosyncratic simplifications is confronted by data which is itself complex, and is therefore likely to acquire a complex language. The simplifying effects of adult learning therefore seem unlikely to offer a complete account for population effects on language complexity. However, in our final experiment, we find that speakers of a more complex language adapt to interlocutors who speak a simpler variant, and those simplifications can persist beyond the course of that interaction. Asymmetric alignment during interaction therefore provides a plausible mechanism explaining how simplifications initially made by a small proportion of language users in a population might spread and exert a disproportionate influence on that population's language, and therefore provides an additional linking mechanism which could explain the observed correlations between population composition and language complexity.

## Supporting information


**Data S1.** Stimuli set for Experiments 1 and 2.
**Data S2.** Experiment 1 complexity: variability of suffixes.
**Data S3.** Experiment 2 input data speakers.
**Data S4.** Experiment 2 complexity: Variability of suffixes.
**Data S5.** Experiment 2 input complexity.
**Data S6.** Stimuli set for Experiment 3.

## Footnotes

1

(1) $$Accuracy\left(p,t\right)=1-\frac{1}{M}LD\left(p,t\right),$$

where *LD*(*p*,* t*) is the Levenshtein distance (Levenshtein, 1966) between the target string *t* and the produced string *p* (i.e., the smallest number of insertions, deletions or replacements required to convert *p* into *t*), and *M* is the length of the longer string of *p* and *t*, therefore normalizing for length.

2 At Round 2, average stem accuracy is 0.84 ± 0.08 (95% CI) and average suffix accuracy is 0.62 ± 0.07. At Round 8, average stem accuracy is 0.97 ± 0.05 and average suffix accuracy is 0.92 *±* 0.05.

3 This includes all models with up to two interactions, though the pattern of results is the same if we only include models with one or no interactions.

4 Note that this measure assigns high complexity to random systems, since the conditioning of affixes in such systems would map to multiple semantic features. For example, if we take the Round 2 language from Tables 2 and 3 and randomize the assignment of affixes within each word class (i.e., re‐assign all the Q affixes to randomly selected Q stems and so on), then we will tend to generate a language with higher complexity: Very few of these randomly shuffled versions of the simple Round 2 system have a system of affix variation where affix choice maps only to Number of the stem. More concretely, the mode complexity of 100 randomized versions of the Round 2 language shown in Table 2 is 3 for Q suffixes, 3 for N suffixes, and 3 for V suffixes.

5 Note that while this is experiment is again concerned with comparatively complex and simple variants of languages, unlike in Experiments 1 and 2 we are not considering inflectional morphology here. The change in the target language for Experiment 3 was for practical reasons. In piloting, we found that training participants on systems with inflectional morphology of varying complexity led to the Simple speaker reaching criteria and then having to wait for a very long time for their partner to do the same, which we considered a substantial potential confound. We found that with our revised method, reported here, both the Simple and Complex speaker were able to reach criteria in comparable times.

6 We carried out a chi‐squared test as a logit mixed model with dyad type as fixed effect and participant identity nested within dyad as a random intercept effect failed to converge.
