# Supplementary material for: Adult Learning and Language Simplification
Source: Cogn Sci. 2018 Oct 15;42(8):2818–54. doi: 10.1111/cogs.12686 (PMC6492256; doi:10.1111/cogs.12686)
Supplement: Supplementary file 1 — Data S1. Stimuli set for Experiments 1 and 2. Data S2. Experiment 1 complexity: variability of suffixes. Data S3. Experiment 2 input data speakers. Data S4. Experiment 2 complexity: Variability of suffixes. Data S5. Experiment 2 input complexity. Data S6. Stimuli set for Experiment 3. [file COGS-42-2818-s001.pdf]

Adult learning and language simplification: supplemental information

Mark Atkinson*[1], Kenny Smith[2], and Simon Kirby[2]

[1]Psychology, University of Stirling, United Kingdom
[2]School of Philosophy, Psychology and Language Sciences, University of Edinburgh, United Kingdom

1 Stimuli set for Experiments 1 and 2

The set of images for Experiments 1 and 2 is shown in Fig. 1.

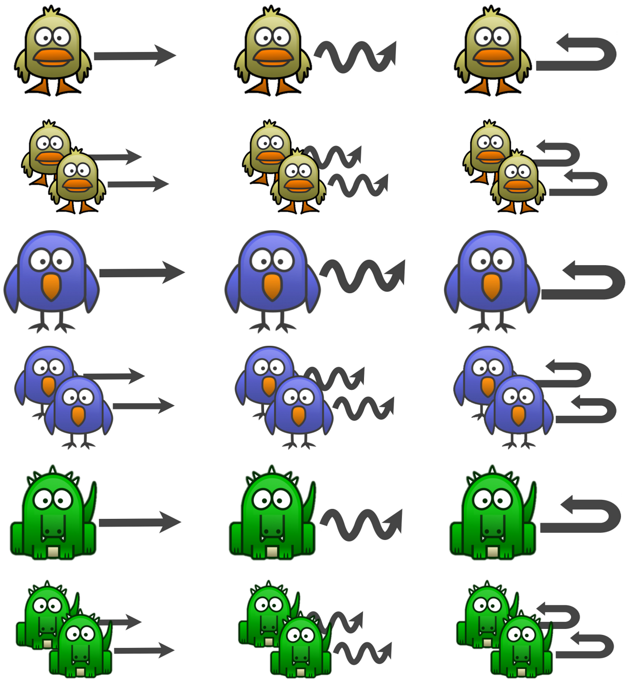

Fig. 1. Stimuli set. Participants were trained on an artificial language which provided descriptions for these 18 scenes, made up of every combination of 3 Animals (duck, bird, and crocodile), 2 Numbers (1 or 2), and 3 Movements (a straight motion, bouncing, and looping).

*mark.atkinson@stir.ac.uk

2 Experiment 1 complexity: variability of suffixes

Our meaning-dependent measure of the complexity of the suffix sets is described in Section 2.2.2 in the main manuscript. Here, we consider an alternative meaning-independent measure of complexity, the entropy of the suffixes for each stem class (Q, N or V). The entropy of a set of signals, $H(S)$, is given by:

$$H(S) = -\sum_{s \in S} P(s) \log_2 P(s) \quad (1)$$

where $P(s)$ is the probability of suffix s. We calculate entropy separately for the suffixes associated with each word type (Q, N, and V): entropy therefore captures the extent to which a single word-type is associated with multiple suffixes, with entropy being low when one suffix is used for most stems (e.g. entropy will be 0 when a single suffix is used consistently for all stems in a category) and high when multiple suffixes are used with equal frequency (e.g. entropy would be 1 if 2 suffixes were used with equal probability). In the target language, entropy for quantifier suffixes ($H(S_Q)$) is 1.918, for noun suffixes ($H(S_N)$) is 0.918, and for verb suffixes ($H(S_V)$) is 2.224. In the Round 2 data shown in Table 2 in the main manuscript, $H(S_Q) = 1$, $H(S_N) = 0.991$ and $H(S_V) = 1.194$ — lower entropy reflects the relative invariance of forms.

Entropy by word type is illustrated in Fig. 2. As can be seen from this figure, entropy for each suffix type converges to the entropy of the target language over rounds; for Q and V suffixes this involves a steady increase in entropy, whereas for the (relatively simple) N suffixes participants over-shoot the target entropy from Round 2, and gradually converge on the target entropy from above.

Entropy scores were submitted to a linear regression (Bates, Maechler, & Bolker, 2013; R Core Team, 2013), with fixed effects of round (revalued such that the model intercept reflects entropy at Round 1), suffix (Q, N or V; this predictor was contrast-coded, such that the model intercept reflects the estimated entropy for the N suffix at Round 1) and their interaction; we included by-participant random intercepts and random slopes for round and suffix. This model confirms that the suffixes differ in their entropy at Round 1, as indicated by a significant intercept (b = 0.977, SE = 0.079, t = 12.290, $p < 0.001$; this simply reflects the fact that N entropy at Round 1 is non-zero) and significant effects for Q and V suffix types indicating that these have higher entropy (Q: b = 0.334, SE = 0.076, t = 4.378, $p < 0.001$; V: b = 0.644, SE = 0.073, t = 8.804, $p < 0.001$). The model also confirms that entropy is stable across rounds for N suffixes (b = -0.007, SE = 0.013, t = -0.505, p = 0.614), but increases for Q and V suffixes (as indicated by significant interactions with round for these suffix types; Q: b = 0.108, SE = 0.015, t = 7.118, $p < 0.001$; V: b = 0.121, SE = 0.015, t = 7.959, $p < 0.001$).

This suggests that the morphological systems produced at Round 2 are somewhat less complex than those at Round 8, in that entropy is lower initially and increases with further

Fig. 2. Average $H(S_Q)$, $H(S_N)$, and $H(S_V)$ (solid lines) and corresponding entropy values for the target language (dashed lines). Entropy increases with training for quantifier and verbal suffixes, but is essentially flat for nominal suffixes. Error bars are 95% confidence intervals.

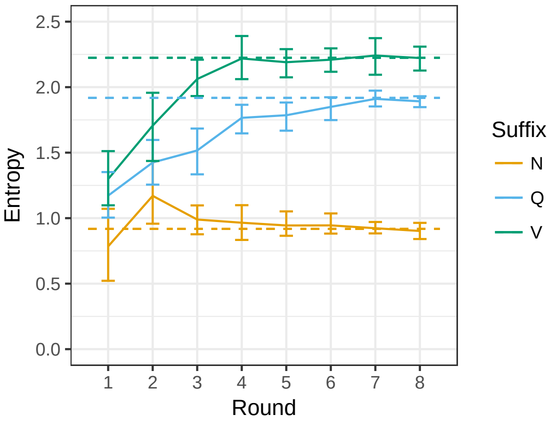

exposure until it approximates the entropy of the target language. This does not appear to be the case for all three word types, however, as the noun suffixes roughly approximate the complexity of the target language from early on and do not show the same increase in complexity. This may be due to the noun system being relatively simple ($H(S_N) = 0.918$, compared to $H(S_Q) = 1.918$ and $H(S_V) = 2.224$): deviations from the target language noun suffixes may be more likely to increase complexity than for the quantifiers and verbs.

3 Experiment 2 input data speakers

This subset of 12 Experiment 1 participants from which the Experiment 2 input is drawn is generally representative of the full set of Experiment 1 participants, with the same trend of an increase in complexity from the Round 2 to Round 8 data. To confirm this, the entropy scores were again submitted to a linear regression (Bates et al., 2013; R Core Team, 2013), with fixed effects of round (revalued so that the model intercept reflects entropy at Round 2), suffix (Q, N or V; this predictor was contrast-coded, such that the model intercept reflects the estimated entropy for the N suffix at Round 2) and their interaction; we included by-participant random intercepts. This differs from the model for the full data set in including no random slope effects. This was necessary for model convergence.

The model confirmed that the suffixes differ in their entropy at Round 2, as indicated by a significant intercept ($b = 0.902$, SE = 0.089, t = 10.142, $p < 0.001$; this reflects the fact that N entropy at Round 2 is non-zero) and significant effects for Q and V suffix types indicating that these have higher entropy (Q: $b = 0.591$, SE = 0.123, t = 4.816, $p < 0.001$; V: $b = 0.829$, SE = 0.123, t = 6.752, $p < 0.001$). The model also confirms that there is no difference in entropy at Round 2 and Round 8 for N suffixes (b = -0.001, SE = 0.020, t = -0.031, p = 0.975), but increases for Q and V suffixes (as indicated by significant interactions with Round for these suffix types; Q: $b = 0.073$, SE = 0.029, t = 2.533, p = 0.014; V: $b = 0.096$, SE = 0.029, t = 3.327, p = 0.001). This pattern of results mirrors

that of the larger set of data from Experiment 1.

4 Experiment 2 complexity: variability of suffixes

Average Round 8 entropy scores by condition are illustrated in Fig. 3. The figure suggests no differences between conditions, an impression which is confirmed by a regression analysis with population size (Large, Small; sum-coded) and input composition (Complex, Mixed; sum-coded) and their interaction as fixed effects and by-participant and by-suffix-type random effects (random intercepts for participant; for the by-suffix-type random effects structure we included random intercepts and random slopes for population size, input composition and their interaction). The fitted model has a significant intercept, again simply indicating non-zero mean entropy (b = 1.633, SE = 0.346, t = 4.719, p < 0.001), but no significant effect of population size, input composition, or their interaction (lowest p observed for the fixed effect of population size: b = 0.046, SE = 0.034, t = 1.350, p = 0.179).

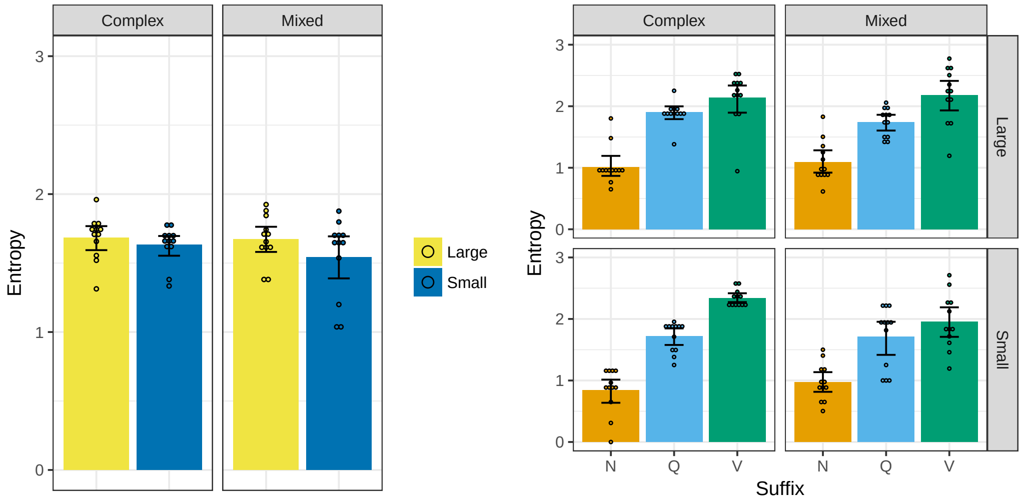

Fig. 3. Entropy by condition and word type in Round 8 of Experiment 2. The left plot shows mean entropy averaging over the 3 suffix types; the right-hand panel shows entropy broken down by suffix. There is no evidence of a condition-dependent difference in entropy. Error bars are 95% confidence intervals. Points illustrate data from individual participants.

5 Experiment 2 input complexity

The same two measures reported for the participant productions (suffix entropy and complexity) can be applied to the input data participants in each of our conditions received. These results are plotted in Fig. 4. As for the output of the participants trained on these various languages, the input languages themselves, despite being composed of rather different constituent languages, show no systematic differences between conditions. This is

confirmed by regression analyses using identical models to those described in the preceding sections, which again show no effects of population size or input composition on entropy (lowest p observed in the fixed effect of input composition, b = -0.023, SE = 0.013, t = -1.765, p = .095, indicating at best a suggestion that overall entropy might be higher in Mixed input data) or complexity (lowest p observed in the fixed effect of population size, b = -1.119, SE = 2.194, z = -0.510, p = 0.610).

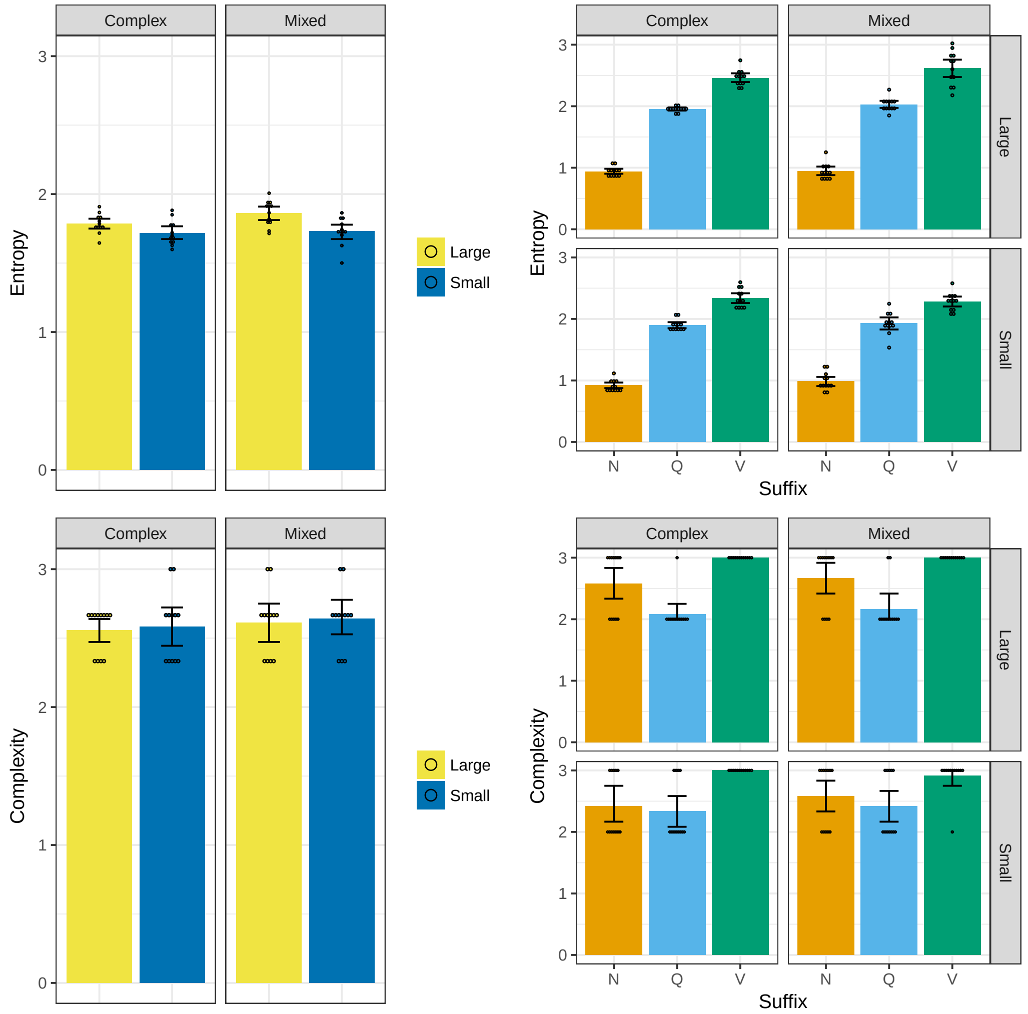

Fig. 4. Entropy (upper row) and complexity (lower row) by condition and word type for the inputs. The left plots shows means averaging over the 3 suffix types; the right-hand panels shows entropy/complexity broken down by suffix. There is no evidence of a condition-dependent difference in entropy or complexity. Error bars are 95% confidence intervals. Points illustrate data from individual participants.

6 Stimuli set for Experiment 3

The set of images for Experiment 3 is shown in Fig. 5.

Fig. 5. Stimuli set. Made up of every combination of 3 Animals (duck, dog, and crocodile) and 3 Movements (a straight motion, bouncing, and looping).

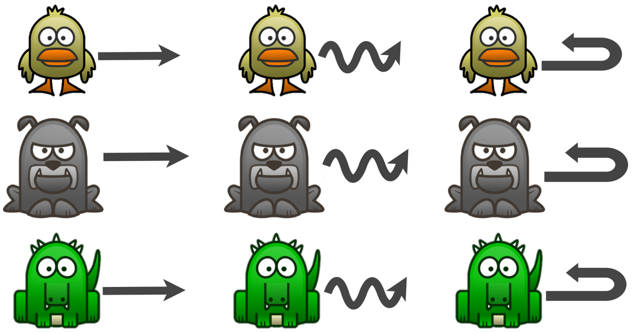

References

Bates, D., Maechler, M., & Bolker, B. (2013). *lme4: Linear mixed-effects models using S4 classes.* Retrieved from `http://cran.r-project.org/package=lme4`

R Core Team. (2013). *R: A language and environment for statistical computing.* http://www.r-project.org/. Retrieved from `http://www.r-project.org/`
